# Towards an Age-Dependent Transmission Model of Acquired and Sporadic Creutzfeldt-Jakob Disease

**DOI:** 10.1371/journal.pone.0109412

**Published:** 2014-10-03

**Authors:** Jesús de Pedro-Cuesta, Ignacio Mahillo-Fernandez, Miguel Calero, Alberto Rábano, Mabel Cruz, Åke Siden, Pablo Martínez-Martín, Henning Laursen, María Ruiz-Tovar, Kåre Mølbak, on behalf of the EUROSURGYCJD Research Group

**Affiliations:** 1 Department of Applied Epidemiology, National Centre for Epidemiology, Carlos III Institute of Health; and Consortium for Biomedical Research in Neurodegenerative Diseases (*CIBERNED*), Madrid, Spain; 2 Carlos III Institute of Health, and Consortium for Biomedical Research in Neurodegenerative Diseases (*CIBERNED*), Majadahonda, Spain; and Alzheimer's Disease Center, Reina Sofia Foundation, Madrid, Spain; 3 Alzheimer's Disease Center, Reina Sofia Foundation, Madrid, Spain; 4 Karolinska Institute, Neurology Division, Stockholm, Sweden; 5 National Hospital, Copenhagen, Denmark; 6 Statens Serum Institut, Copenhagen, Denmark; Nagasaki University Graduate School of Biomedical Sciences, Japan

## Abstract

**Introduction:**

Sporadic Creutzfeldt-Jakob disease (sCJD) might be transmitted by surgery. The purpose of this study was to investigate potential susceptibility to sCJD from surgery at juvenile age and in early adulthood.

**Methods:**

From Danish and Swedish national registries we identified 167 definite and probable sCJD cases with onset from 1987 through 2003, and 835 age-, sex- and residence-matched controls along with their surgical histories. Main, anatomically or etiologically classified surgical procedures followed by a ≥20-year lag were analyzed using logistic regression, and stratified by age at first-registered surgical discharge.

**Results:**

The risk of having a diagnosis of CJD depended strongly on age at first surgery with odds ratio (OR) of 12.80 (95% CI 2.56–64.0) in patients <30 years, 3.04 (95% 1.26–7.33) in 30–39 years, and 1.75 (95% CI 0.89–3.45) in ≥40 years, for anatomically classified surgical procedures. Similar figures were obtained for etiologically classified surgical procedures.

**Conclusions:**

Risk of surgical-acquired sCJD depends on age at exposure; this pattern is similar to age-specific profiles reported for CJD accidentally transmitted by human pituitary-derived growth hormone and susceptibility curves for variant CJD estimated after adjustment for dietary exposure to bovine spongiform encephalopathy. There might be an age-at-exposure-related susceptibility to acquire all CJD forms, including sCJD from routine surgery.

## Introduction

Sporadic Creutzfeldt-Jakob disease (sCJD) is the most frequent neurodegenerative condition among the rare human transmissible spongiform encephalopathies. It has an annual incidence of 1–2 per million and is found worldwide [1]. Other CJD forms include genetic CJD, caused by mutations in the gene encoding PrP (*PRNP*); acquired, ie, accidentally transmitted or iatrogenic CJD (iCJD); and variant CJD (vCJD). iCJD has been reported to be transmitted by neurosurgical instruments used on persons incubating or clinically presenting with CJD, by dura mater grafts, or by treatment with human pituitary hormones. While vCJD has been linked both to dietary exposure to bovine spongiform encephalopathy (BSE) and, more rarely, to transfusion of blood from infected donors, it has not been associated with either general or dental surgery (see reference [2] for a review). Lastly, Kuru, a disease described among the Fore people of Papua New Guinea, has been attributed to ritual cannibalistic practices by natives [1]. The pathogenesis of vCJD transmission by surgery, including neurosurgery in particular, is still being considered. In all forms of CJD, disease progression and cellular death is determined by cell-to-cell transmission of a pathologic isoform (termed PrP^Sc^) of the normal prion protein (PrP^C^), both denoted in amyloid fibril protein nomenclature as APrP [3].

The cause of sCJD is still poorly understood. A number of studies report no association or report even protective effects of surgery, including neurosurgery (see references [2], [4] for reviews). Several of these studies may be subject to bias, and there is increasing epidemiologic evidence of associations to history of general surgery [5], [6], with long incubation periods [6], surgery of the retina and peripheral nerves after shorter incubation intervals [7], and, with less consistent results, to blood transfusion connected to surgery after a >10-year lag [8], [9]. Experimental evidence suggests that infectious PrP^Sc^ bound to the surface of instruments reused during major surgical interventions may transmit CJD despite repeated cleaning and sterilization following previous prion-unaware protocols [10]. Taken together, indicate that a proportion of sCJD may indeed have been accidentally transmitted in origin [5]–[8].

A variation in risk of sCJD in strata by age at clinical onset was reported using matched controls (MCs), unmatched controls, and latency analysis [6]. Excess risk was higher for cases with onset at ages <68 years than for those with onset at ages ≥68 years; these case-groups differed in age at first-registered surgery undergone ≥20 years prior to CJD onset, ie, (mean, SD) 34.0, 7.2 years vs 51.5, 4.2 years, respectively [6]. A plausible interpretation for such variation was the presence of effect modification by surgery undergone at earlier ages, due to age-related biologic host susceptibility.

The aim of the present study, pursuing the same line of investigation as two preceding reports [6], [7], was to use the same database to quantify the variation in surgical risk of sCJD potentially determined by age at surgery.

## Materials and Methods

The study was designed as a registry-based case-control study and has previously been described in detail elsewhere [6], [7]. We included 167 probable or definite sCJD cases who, after being identified from surveillance and death registries, fulfilled established diagnostic criteria, showed clinical onset during the period 1987–2003, and resided in Denmark or Sweden [7]. A population-control set comprising 835 controls matched by gender, year and month of birth, and municipality of residence at death of the corresponding case (5 matched controls (MCs) per case), were randomly sampled from the national study populations aged 40 years and over resident during the abovementioned study period [6], [7]).

Our analysis was restricted to surgical interventions that were followed by long latency periods. Using the body-system classification of surgical procedures (SPs), exposure was defined as first major SP linked to a discharge preceding the clinical onset of illness for cases or the corresponding index date for controls (ID-2) by at least 20 years. We identified main surgical procedures, ie, those SPs remaining after excluding subsidiary procedures (such as punctures, transluminal endoscopic procedures and others), see Table 1 foot notes, associated with hospital discharge preceding the operational time point for onset or ID-2 by ≥20 years. Age at discharge was calculated and assumed to represent age at surgical intervention, disregarding the number of SPs coded per discharge. When the main SPs had been etiologically classified and grouped by presumed risk level using a reported method [7], analyses were performed for presumed *higher-risk* and *lower*-*risk* SPs at the above lag (≥20 years). Conditional logistic regression was used to determine the odds ratio (OR) in three age strata defined by age at first-registered surgical discharge followed by the abovementioned ≥20-year lag, ie, <30, 30–39, and ≥40 years. Results were tabulated and, for purposes of interpretation, those for age-at-first-surgical-discharge strata were plotted on a graph which also showed published results of age-at-exposure assessments for iCJD and vCJD [11]–[13].

**Table 1:** Associations for surgical procedures predating the time point of clinical onset in cases or the corresponding index date in controls by ≥20 years.

Model	Exposure level	Age (years) at first-registered SP	Subject	n	%	OR	(95% CI)
*Model 1* lag of ≥20 y Body-system classification of SPs	Unexposed	All ages	Cases	134	(80.2)	-	-
			MCs	745	(89.2)	-	-
	Exposed to main SPs	All ages	Cases	32	(19.2)	-	-
			MCs	89	(10.7)	2.44	(1.46–4.07)
	Exposed to subsidiary SPs^a^	All ages	Cases	1	(0.6)	-	-
			MCs	1	(0.1)	6.82	(0.41–113)
*Model 2* lag of ≥20 y Body-system classification of SPs	Unexposed	All ages	Cases	134	(80.2)	-	-
			MCs	745	(89.2)	-	-
	Exposed to main SPs	Mean 26.0, range 17.3–29.5	Cases	7^b^	(4.2)	-	-
			MCs	11	(1.3)	12.8	(2.56–64.00)
		Mean 35.5, range 30.7–40.0	Cases	8^c^	(4.8)	-	-
			MCs	21	(2.5)	3.04	(1.26–7.33)
		Mean 49.9, range 40.0–62.0	Cases	17^d^	(10.2)	-	-
			MCs	57	(6.8)	1.75	(0.89–3.45)
	Exposed to subsidiary SPs^a^	All ages	Cases	1	(0.6)	-	-
			MCs	1	(0.1)	7.46	(0.23–242)
*Model 3* lag of ≥20y Etiologic classification of SPs	Unexposed	All ages	Cases	134	(80.2)	-	-
			MCs	745	(89.2)	-	
	Exposed to HR or LR SPs^e^	All ages	Cases	25	(15.0)	-	-
			MCs	58	(7.0)	2.81	(1.62–4.88)
	Exposed to other risk SPs	All ages	Cases	8	(4.8)	-	-
			MCs	32	(3.8)	1.74	(0.76–3.95)
*Model 4* lag of ≥20 y Etiologic classification of SPs	Unexposed	All ages	Cases	134	(80.2)	-	-
			MCs	745	(89.2)	-	-
	Exposed to HR or LR SPs^e^	Mean 26.4, range 23.9–29.5	Cases	5	(3.0)	-	-
			MCs	6	(0.8)	13.20	(2.47–70.50)
		Mean 36.0, range 30.9–39.8	Cases	6	(3.6)	-	-
			MCs	12	(1.4)	3.57	(1.26–10.10)
		Mean 50.0, range 40.0–62.0	Cases	14	(8.4)	-	-
			MCs	40	(4.8)	2.10	(1.05–4.17)
	Exposed to Other risk SPs	All ages	Cases	8	(4.8)	-	
			MCs	32	(3.8)	1.79	(0.78–4.09)

Models 1 and 2, anatomic SP classification; models 3 and 4, etiologic SP classification. Other risk SPs encompass three reported etiologic SP categories, i.e., *lowest-risk*, *no-risk*, and *not reclassified* [7]. Age subcategories <30, 30–39, and ≥40 years at first-registered surgery.

a Subsidiary procedures' is a heterogeneous category that includes minor surgery (punctures, needle aspiration or biopsy, superficial incisions), other non-surgical, potentially invasive procedures, such as transluminal endoscopies (with or without biopsy), and, in a few instances in Denmark, blood transfusion.

SP-code distribution by body system groups in exposed cases.

b n = 15. Female genital organs and obstetric SP, 7; Digestive system and spleen, 3; Other groups, 5.

c n = 15. Female genital organs and obstetric SP, 8; Digestive system and spleen, 3, Other groups, 4.

d n = 37. Female genital organs and obstetric SP, 18; Digestive system and spleen, 8; Peripheral vessels and lymphatic system, 5; Other groups, 6.

e HR or LR SPs: acronym for *higher-risk* or *lower-risk* SPs [7].

Scientific evaluation and preliminary ethical clearance of the research proposal was done by the EU Research Commission's “Concerted Action QLRG3-CT-2002-81223”. The study was formally notified to the Danish Data Protection Agency (record no. 2003-41-3104) and approved by the Karolinska Institute Ethics Committee (South; report no. 452/02, 2002-12-02) and "Regionala Etiskprövningsnämnden" dnr 04-171T 2004-04-29 1). Written consent was not given by patients for their information to be stored in the hospital data base and used for research since it was not needed. In Sweden, patients were never personally identified after register linkage took place by registries administrative officers. A data base was built after substituting at registries level each personal identification number by serial individual numbers. Data stored in official Swedish registries were used following legal regulations. The study conforms with Danish legislation requiring data management after notification to the Danish Data Protection Agency (Act on Processing of Personal Data - Act No. 429 of 31 May 2000). According to the Danish legislation, there is no need for scientific-ethical clearance of registry based studies, nor is there a need for written consent by cases (Act nr 593 of 14/06/2011, Section 10 paragraph 2). Additionally all CJD patients had died by the time of data collection. No biological or tissue data was studied. The dataset is available on request to last author.

## Results

**Figure 1:**
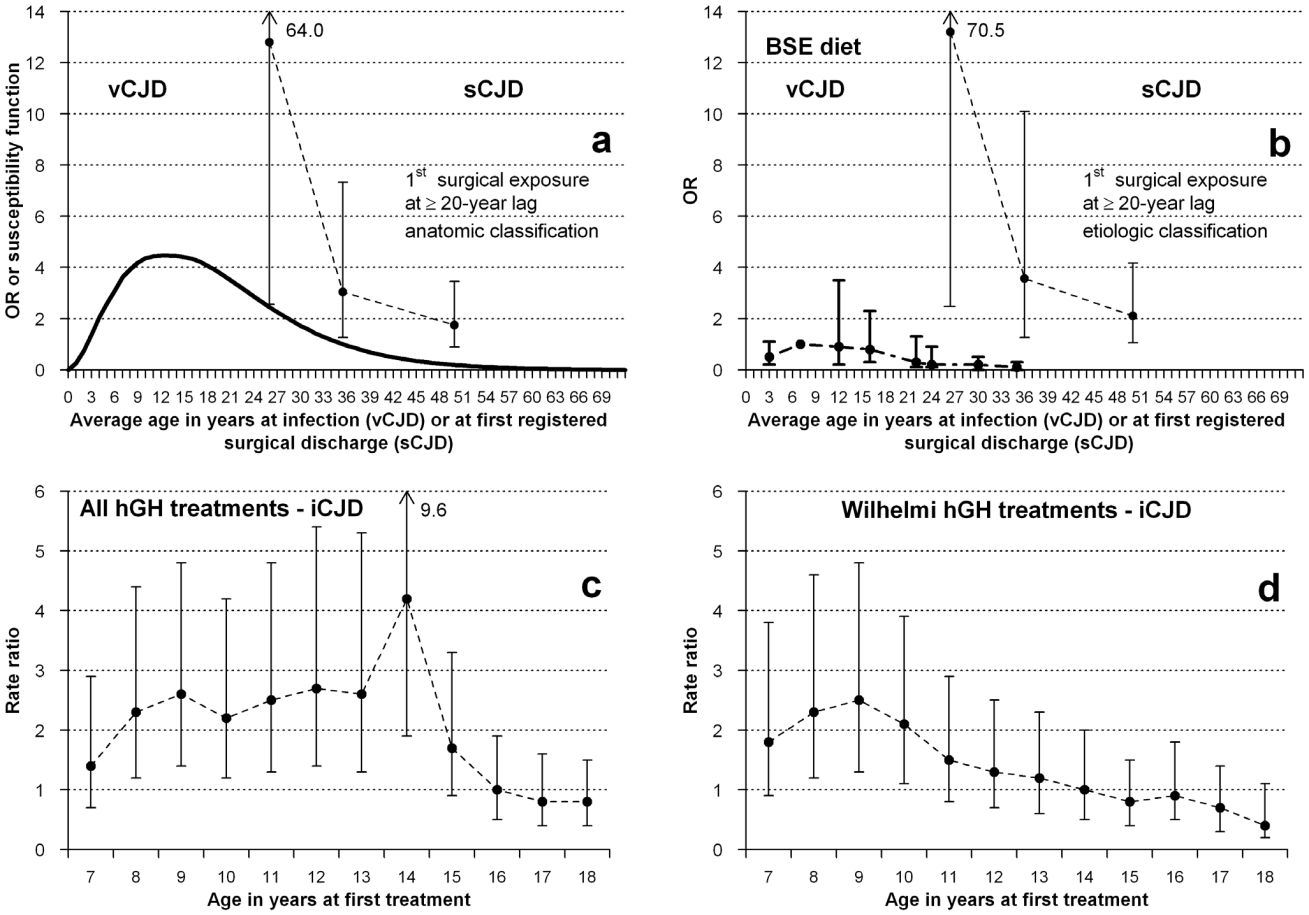
Potential age-at-exposure-related susceptibility for the following different CJD forms and exposures: (a) Age-susceptibility function for vCJD in the UK [12], and results from Table 1 for risk of sCJD from age at first hospital discharge associated with a registered, main surgical procedure, at a lag of ≥20 years. (b) Reported estimated risks relating to 5- to 10-year age groups, after adjustment for dietary exposure to bovine material and average incubation period established at 12.6 years for variant CJD in the UK [13], and plotted results from Table 1 for risk of sCJD from age at first hospital discharge associated with an etiologically reclassified *higher-risk* or *lower-risk* procedure, at a lag of ≥20 years. (c) Reported rate ratios of accidentally transmitted CJD (iCJD), for ever treatement vs never treatement with pituitary growth hormone (all treatments) at specific ages [11]. Reference: all other ages at treatment. (d) Reported rate ratios of accidentally transmitted CJD (iCJD) for ever treatment vs never treatment with pituitary growth hormone processed with the Hartree-modified Wilhelmi method (an hGH preparation associated with highest risk of iCJD among hGH-treated cohorts) at specific ages [11]. Reference: all other ages at treatment.

This analysis included 123 study participants, made up of 33 cases, and 90 MCs with a record of surgery and documented age at discharge. The remaining 879 persons included in the database, 134 cases and 745 MCs, were free of such surgery. History of surgery followed by a ≥20-year lag was associated with significant excess risk of sCJD, with similar estimates for anatomically and etiologically classified SPs (ORs of 2.44 and 2.81, respectively; see Table 1). The risks at this latency for age at first-registered surgical discharge for main anatomically classified SPs yielded ORs of 12.80, 3.04, and 1.75 when comparing persons first exposed at ages <30, 30–39, and ≥40 years, respectively. A similar trend, with higher risk in the lowest age-category, was seen when etiologically reclassified *higher-* and *lower-risk* procedures were compared (ORs of 13.2, 3.57, and 2.10 for ages <30, 30–39, and ≥40 years, respectively). The patterns obtained in our study suggest an increasing risk with decreasing age at first-registered surgery, particularly in the case of the <30-year age stratum. The ORs for each of three categories of age at first-registered surgery at a ≥20-year lag for main SPs, specified in Table 1 are graphically shown in Figure 1.

A complementary analysis using an alternative set of models, with categories of age at first surgical exposure with a 20-year lag in quintiles, yielded similar results. For the lowest quintile, the OR for anatomically classified main procedures, with 8 exposed cases and 17 exposed MCs, mean age 27.5 years, was 5.15 (95% CI 1.58–16.8). For etiologically classified main SPs, with 5 exposed cases and 12 exposed MCs, mean age 28.5 years, the OR was 3.53 (95% CI 0.88–14.3). Mean ages for the lowest quintile were higher than for the corresponding <30-year group.

## Discussion

This study represents the fourth sequential approach to quantifying associations between surgery and sCJD, using unique material and purpose-designed methods [6], [7], [14]–[16]. Study strength is indicated by the systematic use of registries, which allowed for less-biased assessment of exposure variables (SPs) and for validated outcomes [17]. Interpretation of results may have been affected by methodologic limitations, including lack of control of confounding due to blood transfusion (BT). The fact that the frame of biological plausibility for a causal interpretation of results is limited, owing to the relative infrequency of neurosurgical procedures in this dataset (only one such SP, peripheral nerve surgery due to amputation, among cases was included) does not preclude inference for general surgery.

### Excess risk in age-at-first-registered-surgery strata

We found an age-at-exposure risk pattern which mimics that reported for vCJD. This is consistent with the possibility of an age-related common biologic-etiologic determinant of risk for different CJD forms, possibly neglected until now. This pattern is in agreement with the currently accepted notion of susceptibility, ie, different effects from a given exposure [2], [18]. Since SP registration was first introduced in 1970 in Denmark and in the early 1970s in most Swedish counties [19], effects observed by our study among those exposed earliest in life might have incorporated risk acquired from unregistered surgery undergone at even lower ages.

Attributing an age-at-exposure differential excess risk to age-at-exposure susceptibility would require that the nature of surgical exposure or preventive practices did not vary with age. The weight of reproductive surgery was similar across age groups. Additionally, excess risk of reproductive-organ surgery was similar to that seen for other types of surgery, such as gastrointestinal or orthopedic, at the same latency [6]. Tonsillectomy, of which rates are known to vary by age was unfrequently registered, i.e. in two controls at ages 26 and 56 years. One case had tonsil incision at 28 years. Such unprecise data would correspond to a fivefold higher proportion of tonsil-related surgery among cases in the youngest age-group. It is nonetheless likely that other cases and controls underwent tonsillectomy at >20-year lag as outpatients. This underascertainment of exposures represents a limitation. Based on the same material, we previously estimated a modest and unprecise excess risk for surgery related to the tonsils OR 3.59 (95% CI 0.39 to 32.8) [7]. Recapitulating, results for tonsillectomy are not fully in line with the high risk for surgery at ages 23.9–29.5 years seen in the present study; this can be ascribed to low numbers but it is also possible that hospital cleaning and disinfection practices, including increase use of single-use materials, have improved in recent decades, when registered surgery at youngest ages was most frequent. Such an expected effect will tend to dilute our main finding, i.e., a higher risk among the young, and could, in addition to the issue of low numbers, explain the previous observation on procedures related to the tonsils [7].

### Age-related susceptibility to exposure effects in other CJD forms

Age-related susceptibility to the effects of dietary exposure to BSE in the United Kingdom (UK) constitutes a key etiologic and epidemiologic feature of vCJD [12], [13] supported by the recently suggested role of the phosphatidylinositol pathway [20]. As can be seen in Figure 1-a and 1-b, reported data on age-specific risk of vCJD in the UK, whether estimated as a susceptibility function or calculated by Böelle et al. after adjustment for age-specific magnitude of dietary exposure to BSE, approximately depict log-normal functions [12], [13]. Additionally, a variation in risk of iCJD by age at treatment with cadaveric pituitary-derived growth hormone (hGH), ie, ever versus never treated in each age category, has been suggested from a descriptive report on iCJD incidence rates in UK cohorts, see Figure 1-c and 1-d [11]. The highest rates were seen among persons treated at age nine years with hGH prepared by the Wilhelmi method of extraction, and at later ages, for all hGH treatments [11]. When these data were plotted on Figure 1, a tantalizing visual interpretation suggested that the iCJD curve matched the vCJD risk-function profile at its highest-risk age interval. Age-related susceptibility for iCJD due to dura mater grafts has not been suggested despite many iCJD patients received grafts at young age with long survival, for instance associated with posterior fossa surgery on malformations. The high ratio of approximately one iCJD case per 2000 grafts [21] indicates that there is room for age-related susceptibility for iCJD due to dura mater.

On the basis of the above limited, though detailed, data for iCJD, sCJD, and vCJD, a general model of age-related susceptibility to CJD could be hypothesized.

### Surgical history and risk of sCJD

A recent review of 18 case-control studies which examined links between medical procedures, particularly surgery and blood transfusion, and sCJD, identified many other associations, including protective ones, and highlighted diverse sources of potentially underlying bias [2]. The review concluded that potential methodological pitfalls in case-control studies on surgery and risk of sCJD were (a) the use of hospital controls and sampling controls close to or at the end of long case incidence periods, (b) exposure assessment in different lifetime periods for cases and controls and (c) potential confounding by concurrent procedures, particularly surgery and intra-operative blood transfusions. Three types of positive associations with decreasing biological plausibility were proposed. a) An increased frequency of surgery during the prodromal phase or early clinical course in sCJD was considered to be a stressful trigger of sCJD [22] or, alternatively, attributed to reverse causality by prodromal or subclinical disease [14]. b) Positive associations for SP and blood tranfusions, with the strongest evidence of an association based on latency analysis at >10- or >20-year lag, were consistent with the protracted incubation periods in human prion disease, but might have involved organs or tissues that are not known to be infectious in sCJD [9], [10]. Although case-control studies must be interpreted with caution and there may be confounding of SP by BT, the positive findings in relation to SP and BT may indicate a true risk for sCJD c) As a third explanation, specific procedures at a shorter latency could be a risk factor, e.g. retinal surgery with about a mean 10-year lag or coronary surgery in the decade preceding sCJD onset. The interpretation of these findings required an assessment of specific, speculative hypotheses. Possibilities include the direct spread of prions to the retina, or the confounding by risk factors shared by coronary disease and sCJD [6], [14]. For details we refer the reader to our abovementioned review.

### Public Health implications

We have previously estimated a population-attributable proportion of surgically transmitted sCJD in Denmark and Sweden of 18%. This estimate was based mainly on surgery performed on middle-aged and elderly patients [6]. If susceptibility is age-related, as the present analysis indicate, the population-attributable fraction was underestimated. This idea is based on the fact that under-registration of SP prior to 1970 was highest for those first exposed at ages <30 years, and that positive life-time surgical history in the UK at these ages reaches 60% [23]. Hence, risk of sCJD might be highest for ages at surgery not completely covered by our study. Estimating the public health impact would require a study update to be conducted after a 10-year interval, ie, at the present, to cover surgery at lower ages and infrequent, *high-risk* procedures such as neurosurgery.

Our results may imply that written recommendations for prevention, such as the need for single-use equipment, and for organizational measures may be particularly relevant for young surgical patients [24]. In addition, middle-aged and elderly patients inadvertently exposed to potentially contaminated instruments might not qualify as "at-risk persons for public health purposes", in view of their lower risk of acquiring sCJD. The results of this study would also support the need for EU Member States to implement continued surveillance of and public health research into all CJD forms, and the recording of patients' complete surgical histories.

To sum up, the results of this study suggest that, in line with reported findings for iCJD and vCJD, there is an age-at-exposure-related susceptibility for risk of sCJD from routine surgery. This observation is relevant for epidemiologic research, clinical guidance to prevent CJD transmission in medical settings, and CJD surveillance.
